# Alternative Motor Task-Based Pattern Training With a Digital Mirror Therapy System Enhances Sensorimotor Signal Rhythms Post-stroke

**DOI:** 10.3389/fneur.2019.01227

**Published:** 2019-11-22

**Authors:** Chao-Sheng Chang, Ying-Ying Lo, Chien-Liang Chen, Hsin-Min Lee, Wei-Chi Chiang, Ping-Chia Li

**Affiliations:** ^1^Department of Healthcare Administration, I-Shou University, Kaohsiung, Taiwan; ^2^Department of Emergency Medicine, E-Da Hospital, Kaohsiung, Taiwan; ^3^Department of Physical Therapy, I-Shou University, Kaohsiung, Taiwan; ^4^Department of Occupational Therapy, I-Shou University, Kaohsiung, Taiwan

**Keywords:** mirror therapy system, occupational therapy, pattern training, sensorimotor training, stroke therapy

## Abstract

Mirror therapy (MT) facilitates motor learning and induces cortical reorganization and motor recovery from stroke. We applied the new digital mirror therapy (DMT) system to compare the cortical activation under the three visual feedback conditions: (1) no mirror visual feedback (NoMVF), (2) bilateral synchronized task-based mirror visual feedback training (BMVF), and (3) reciprocal task-based mirror visual feedback training (RMVF). During DMT, EEG recordings, including time-dependent event-related desynchronization (ERD) signal amplitude in both mu and beta bands, were obtained from the standard C3 (ispilesional hemisphere, IH), C4 (contralesional hemisphere, CH), and Cz scalp sites (supplementary motor area, SMA). The entire ERD curve was separated into three time-phases: P0 (−2 to 0 s), P1 (0 to 2 s), and P2 (2 to 4 s). Four-way and subsequent repeated-measures analyses of variance were used to examine the effects of group (stroke vs. control group), test condition (NoMVF, BMVF, and RMVF), time-phase (P0, P1, and P2), and brain area (IH, CH, SMA) on the ERD areas (%) in mu and beta bands. For the mu band, generally, ERD areas (%) were larger in the control than in the stroke group. The ERD areas (%) were largest under the RMVF condition, followed by BMVF and NoMVF conditions. Similar results were found in the beta bands. The main effects of group, time-phase, and test condition on the ERD areas (%) were significant for the three brain areas, except the main effect of group in the SMA (Cz) and CH (C4) brain area. The ERD areas (%) were larger in the control than in the stroke group. The ERD area (%) was significantly larger during P1 than during P0 and P2 (*ps* < 0.02), and during P2 than during P0 (*ps* < 0.01). The ERD area (%) under the RMVF condition was significantly larger than that under the BMVF condition and NoMVF condition (*ps* < 0.05). The present study suggests that cortical activation particularly in the SMA (Cz) of the brain increases in the RMVF condition in both healthy subjects and stroke patients. This result supports the hypothesis that stroke patients may benefit from RMVF training.

## Introduction

The number of stroke patients living with motor impairments is a significant public health concern. Upper extremity complications are common following stroke and may be seriously debilitating, causing the stroke patient to experience difficulties with activities of daily living (1, 2). To support and improve quality of life in stroke patients, various motor-assistance devices and new treatment methods have been developed to restore motor functioning in stroke patients (3–5). In particular, various treatment methods for upper extremity rehabilitation have emerged; these include virtual reality, robot-arm training, mental practice, and mirror therapy (MT) (6–11).

MT has been used to alleviate phantom limb pain and facilitate limb recovery in stroke patients (12–14). MT is a cost-effective and simple alternative method for stroke rehabilitation. It involves creation of a reflective illusion of the affected limb with an action-observation technique. MT provides visual feedback impressions created by observing the image of the less-affected upper extremity projected over the affected limb. The mechanisms underlying the efficacy of MT include its facilitation of motor learning, promotion of interhemispheric communication and balance between the motor cortices, and increased cortical activation after stroke (3, 6, 12, 15, 16). Likewise, mirror visual feedback is thought to restore cortical reorganization, and thereby to improve motor recovery in weak or paralyzed limbs.

In our previous study (17), we developed a new digital mirror system to address the limitations of traditional MT. Compared to traditional MT, our system has three distinct features: it provides (1) vivid, high-resolution MVF in a first-person perspective, (2) an improved viewing angle for the mirror visual feedback (MVF) on the front screen that produces less tension in subjects' cervical posture, and (3) three training conditions: no MVF training (NoMVF), bilateral synchronized task-based visual feedback training (BMVF), and reciprocal task-based visual feedback training (RMVF). Moreover, the program with RMVF was modified to serve as a task-oriented program with reciprocal movement to allow repetitive practice that facilitates motor recovery and bilateral motor coordination in stroke patients.

Our prior work primarily examined the effects of RMVF on cortical activation using the event-related desynchronization (ERD) mu rhythm. The ERD mu rhythm occupies an EEG frequency range of 8–12 Hz and is recorded from sensorimotor cortical areas (18). The ERD in the mu frequency bands during action observation or execution may reflect indirect modulation of motor cortex activity, as mu rhythms are suppressed by mirror neurons. The results of our previous study revealed that RMVF was reinitiated and prolonged with power changes in the ERD mu rhythm.

Moreover, oscillations in the beta frequency band (15–30 Hz) are known to be important in movement (19). Several studies have used electroencephalography (EEG) beta band to reflect movement-related processing results in the modulation of neuronal synchronization. For examples, the study by Fong et al. used ERD in mu and beta bands to examine whether recruitment of the mirror neurons, mediated recognition of the mirror visual feedback during MT in stroke patients and health controls (20). The study by Jeunet et al. have developed an EEG-based (including mu and beta bands) brain computer interface and neurofeedback targeting sensorimotor rhythms to improve motor skills. Accordingly, mu and beta bands could be proper indices when examining the MT effectiveness (21).

To extend our previous research, we here evaluated the feasibility of different types of visual feedback using the DMT system to activate the cortical somatosensory areas with ERD patterns in adult stroke patients. We hypothesized that training modality under the RMVF condition by using DMT system would enhance the effects of motor training on cerebral activation mu and beta in stroke patients.

## Materials and Methods

### Experimental Subjects

Two groups of subjects (a stroke group and a control group) were allocated to three different visual feedback conditions with the task-based DMT system. Based on sample size calculation (performed with G-power software, with power set at 0.8, α = 0.05, and effect size = 0.25), total sample size above 28 was appropriate. We recruited 32 individuals (16 per group) for the study. Participants with stroke were recruited from the outpatient rehabilitation clinic of a medical center. Inclusion criteria for the stroke group were as follows: (1) age 20–65 years, (2) duration since stroke onset more than 3 months, (3) right-handedness with left hemisphere stroke (confirmed by computed tomography or magnetic resonance imaging scans), (4) ability to understand instructions, (5) Mini-Mental State Examination scores above 20, (6) upper limb motor score on the Fugl-Meyer Assessment >22, and (7) Brunnstrom stage 5–6. Subjects were excluded if they had (1) participated in other rehabilitation-related or therapeutic medicine studies within 3 months of this study, (2) a history of orthopedic or neuromuscular diseases or neurological complications (e.g., upper limb fractures or peripheral nerve injury and the occurrence of apraxia), (3) severe visual or visual perception impairments (e.g., neglect or reduced visual field), or (4) had psychiatric comorbidities. Additionally, individuals were excluded if they were unable to cooperate with the researchers due to cognitive or personality limitations or were unwilling to sign a consent form or wear an EEG cap. The mean ages of the control group and the stroke group were 50.813 (*SD* = 8.635) and 54.375 (*SD* = 10.911) years old. No significant age difference was found between the two groups (*p* = 0.314). Table 1 shows the general characteristics of the stroke patients.

**Table 1:** Characteristics of study participants.

**Participant**	**Age (years)**	**Sex**	**Lesion location and etiology**	**Time since stroke (months)**	**FMA-UE**	**MMSE**
P1	61	M	Brainstem hemorrhage	15.7	50	26
P2	52	M	L-MCA infarction	7.2	53	24
P3	47	M	L-Corona radiata hemorrhage	12.4	55	27
P4	69	F	Pons/L-Corona raditata infarction	11.6	43	25
P5	42	M	L-PCA/Basilar artery infarction	24.3	54	29
P6	49	M	L-ICA infarction/Posterior putamen lesion	3.7	52	30
P7	30	F	L-AVM rupture with deep intraparenchymatous hematoma	16.13	56	28
P8	46	M	L-Posterior internal capsule infarction	9.18	51	30
P9	63	F	L-MCA infarction	54.13	53	30
P10	58	M	L-Basal ganglia hemorrhage	21.6	52	27
P11	70	F	L-ICA infarction	27.2	48	26
P12	60	M	L-Basal ganglia hemorrhage	73.8	49	25
P13	51	M	L-Corona radiata infarction	20.27	51	28
P14	68	M	L-MCA infarction	51.14	50	30
P15	47	F	Brainstem hemorrhage	114.80	52	30
P16	57	F	Basilar artery infarction	30.30	45	30

*FMA-UE, Fugl-Meyer Upper Extremity Assessment; MMSE, Mini-Mental State Examination; MCA, middle cerebral artery; PAC, posterior cerebral artery; ICA, internal carotid artery; AVM, arteriovenous malformation; L, left*.

The protocol was approved by Kaohsiung Cheng Kung Memorial Hospital and E-DA Hospital ethics committee; the clinical trials approval certificate numbers are 106-1356C and EMRP-104-013. All participants provided written informed consent.

### Experimental Design

The experiment was designed to allow between-subjects comparison of the stroke and control groups. The ERDs of stroke patients and healthy controls during bimanual and reciprocal coordination movements were compared. All experiments were conducted in the same bright room with air conditioning to maintain a stable air temperature. Participants were seated in a height-adjustable chair, with their forearms resting on the therapy table. They were asked to perform two types of motion (bilateral task-based and reciprocal action task-based with a paper cup) repeatedly or to perform no motion. Given that time factors (duration and interval) might affect mu or beta rhythms' ERD recovery speed, participants were trained to self-pace their task-based movements so that they could maintain a duration of 1–2 s and an interval of 15 s. Before the experiment, subjects were instructed to relax and place both hands on a desk.

### The Digital Mirror Therapy System (DMT System)

As previously described (17), the Digital Mirror Therapy system (DMT system) consists of a host personal computer (PC), camera, and therapy table with a movement area and a mirror area. The participant was asked to sit behind the table and place a button and their non-impaired hand (active hand) on the movement area. In the mirror area, a slim, 27-inch liquid crystal display monitor with a resolution of 1920 × 1080 pixels (Model IPS277L-BN; LG Inc., Seoul, Korea) was situated slightly above the table to reserve the underlying space for the impaired, non-active hand. Continuous images of the movement area were recorded with a video camera (C920, resolution: 1920 × 1080 pixels; 30 frames per sec; Logitech Inc.), which was positioned above the area. Images were sent to the host PC, which processed the vertically mirrored and/or time-delayed images with a button task-based training pattern.

**Figure 1:**
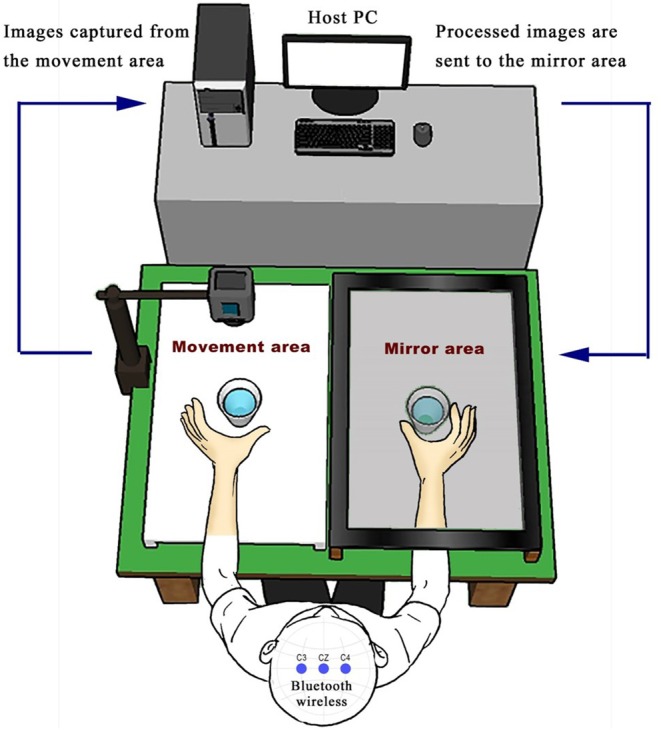
Experimental set up: The DMT system consists of a host personal computer, camera, and therapy table with a movement area and a mirror area. The participant sits behind the table and places a paper cup for task-based training on the mirror area and their non-impaired hand (active hand) on the movement area. In the mirror area of the table, a slim, 27-inch liquid crystal display monitor was situated slightly above the table to reserve the underlying space for the impaired hand as the non-active hand.

**Figure 2:**
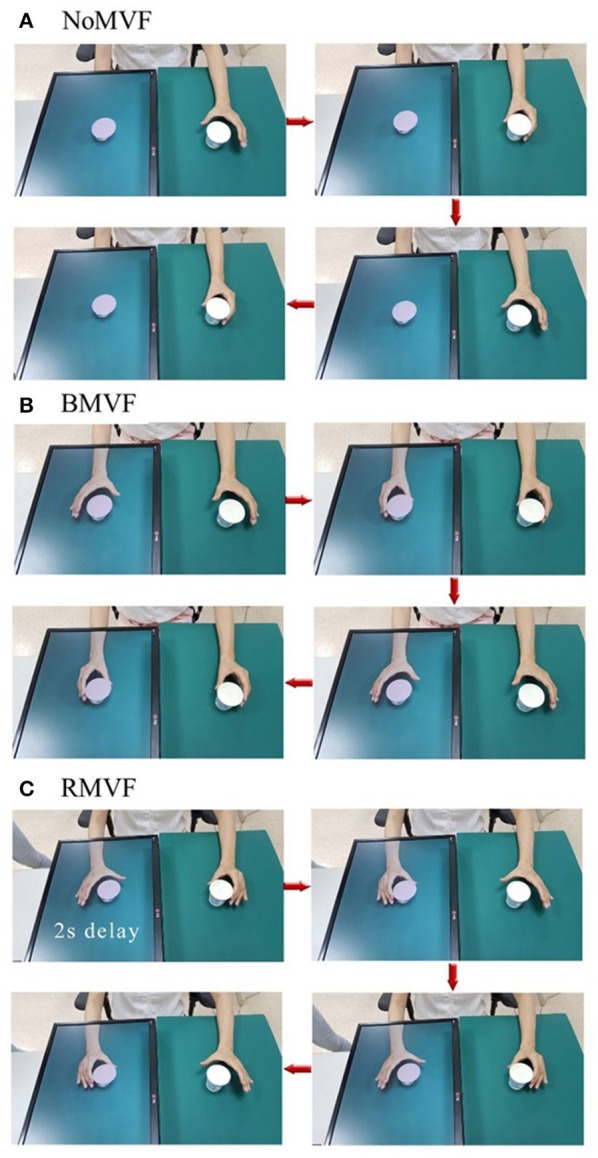
Operation process of three test conditions that were used to test their impact on sensorimotor cortical activity. **(A)** The mirror area showed only a paper cup with a still image of the mirror area without the hand (NoMVF condition). **(B)** The mirror area showed synchronous, simultaneous mirrored images of the movements of the left hand with bilateral task-based mirror visual feedback (MVF; BMVF condition). **(C)** The mirrored images of the movements of the left hand were shown in a 2-s delay of the reciprocal action task-based MVF (RMVF condition).

We modified the previously described (17) protocol using the DMT system (Figure 1). Subjects from the two groups were instructed to place their left hand or unaffected hand in front of the camera, providing a reflection as affected hand. The three different visual feedback conditions for the task-based paper cup grasping tasks were used to test the impact of the different conditions on sensorimotor cortical activity. Under the first condition, the mirrored area contained only a paper cup, with still images of the mirror area without the hand (the “NoMVF” condition). Under the second condition, the mirror area showed synchronous, simultaneous mirrored images of the left hand's movements during a bilateral task-based MVF (the “BMVF” condition). Under the third condition, mirrored images of the left hand's movements were shown with a 2 s delay for the reciprocal action task-based MVF (the “RMVF” condition) (Figure 2). Each condition was performed for 50 trials consecutively.

### EEG and EMG Measurements

A BR Plus Bluetooth wireless 2.1 telemetry system (Brain Rhythm Inc., Zhubei, Taiwan) was used for EEG recordings, which were obtained from seven dry, non-prep spring-load sensor specification electrode pairs (15 × 15 × 12 mm and 200–500 KΩ impedance). Three electrode sensors were placed on the standard C3, C4, and Cz scalp sites, per the international 10–20 system, and were fixed to the center of the movement area. These Cz, C3, and C4 electrode pairs (inter-electrode distance: 2.5 cm) covered the SMA (22) and the ispilesional (active hand) and contralesional (non-active/mirrored hand) primary sensorimotor hand areas. Electrode impedances were checked using an impedance meter (EIM-107 Prep-Check Plus; General Devices Inc., Indianapolis, IN) and were maintained below 5 kΩ. The reference electrode was placed on the right ear lobe. To prevent contamination of recordings by ocular artifacts, a vertical electrooculogram (EOG) was also obtained with a pair of electrodes (Medi-Trace 100; Kendall Inc.) placed above and below the left eye. Recorded EEG signals were sent to a miniature amplifier (Model QP511; Astro-Med, Inc., West Warwick, RI) with a gain of 20,000 and a band-pass filter of 0.12–125 Hz.

For EMG measurements, we modified the BR plus system's Fp1, Fp2, O1, and O2 to EMG signal channels and custom-made a square button that was fixed to the front and back of the scalp overlying the target area of the cortex. A force signal was used to conform in real-time when task-based training patterns occurred with the expected durations and intervals. Two pairs of surface electrodes were placed in parallel on the corresponding brachioradialis belly of the forearm flexors of the active and non-active hand to record EMG signals during training. Finally, EEG, EOG, force, and EMG signals were sent to the PC for further analyses via a data acquisition device (PCI-6259; National Instruments Inc., Austin, TX) with a sampling rate of 1,000 Hz. Brain Viewer (the gallant lab, University of California, Berkeley) was used for basic real-time signal processing, its main function was to acquire raw EEG data outputs, such as the time/frequency-domain signal display, band-pass filter, and LabStreamingLayer broadcasting function. The experimenter was also able to monitor all data on-line using a monitor to confirm its quality, thus allowing for additional grasping trials to be requested if any trials failed.

### Analyses of ERD in Mu and Beta Bands

To retrieve ERD epochs in mu and beta bands from the EEG signal, EMG signals recorded from the active hand were sequentially processed with band-pass filtering (30–300 Hz), band-reject filtering (60, 120, and 180 Hz), rectification, and low-pass filtering (3 Hz). By visually marking the point at which the processed EMG signals increased (considered to be the 0-s point for each grasp, EEG epochs of −2 to 4 s were extracted and further analyzed for each EEG channel. We selected 8–12 and 15–25 Hz as the frequency band in which mu waves and beta waves occurred, respectively, and followed standard ERD processing procedures, as suggested by Pfurtscheller and da Silva (23). An inter-trial variance method was used to remove any possible contributions of phase-locked event-related potentials. Briefly, 50 raw EEG epochs were band-pass filtered (8–12 and 15–25 Hz), had their mean trends removed, were squared for each data point, and were finally averaged across all epochs to obtain a mu or a beta rhythm power amplitude signal. Furthermore, the relative power of the ERD waveform was calculated using the following formula:

$$\begin{array}{l} ERD\left(\%\right)=\left(A-R\right)/R\text{ x }100, \end{array}$$

where *A* and *R* represent the mu or the beta rhythm power amplitude and the reference level, respectively.

A previous study (17) revealed that the mu ERD commences approximately 2 s prior to the onset of a voluntary self-paced finger movement, suggesting that power amplitudes at around 2 s should be used as a reference. In the current study, most subjects exhibited higher mu rhythm power amplitudes 1.8–2.2 s before the grasp movement in the NoMVF condition. We thus chose to assess averaged power amplitudes at around −2 s (−2.125 to −1.825 s of the EEG epoch) in the NoMVF condition as a reference (*R*) for all conditions. To smooth the data and reduce variability, time averaging with a 250-ms window was also adopted. To quantify cortical activation during the period from −2 to 4 s surrounding the grasp movement, we used the averaged area of the entire ERD curve under the reference level as the amplitude parameter for cerebral activation. Furthermore, we separated the entire ERD curve into three time periods: P0 (−2 to 0 s), P1 (0 to 2 s), and P2 (2 to 4 s). For each phase, the averaged ERD area (%) was used to reveal the phasic amplitude of cerebral activation and any time-course changes during the different conditions with task-based grasping of the paper cup.

Off-line processing of force, EMG, EEG, and ERD data was completed by using a custom -made MATLAB program (ver. 7.0; The Math Works Inc., Natick, MA).

### Statistical Analyses

Statistical analyses were performed using SPSS ver. 18.0 (IBM, Chicago, IL). We respectively, examined the effects of group, brain area, test condition, and time-phase on the ERD areas (%) in the mu band (8–12 Hz) and beta band (15–25 Hz). Four-way repeated-measures analyses of variance (ANOVA) was used to assess the interactions and the main effects among four variables (group, brain area, condition, and time-phase). The within-factors included brain area (IH, CH, SMA, recorded from C3, C4, Cz electrodes), condition (NoMVF, BMVF, and RMVF), and time-phase (P0, P1, and P2), while the between-factor was group (the stroke group and the control group). Significant models were assessed by Bonferroni *post-hoc* tests to compare differences between each pair of the four variables.

Moreover, in order to compare the results of this study with those of our previous study, for each group, we replicated the analyses of the previous study. Two-way repeated-measures ANOVAs were used for each brain area to examine the main effects and interactions of the test conditions and time-phases. One-way ANOVA was used to compare differences in the ERD areas (%) under the three conditions at each time-phase for the three brain areas. One-way ANOVA of the overall ERD area (P0 + P1 + P2) was used for each brain areas to examine the main effects of the test conditions. Significant models were assessed by Bonferroni *post-hoc* tests to compare differences between each pair of the four variables.

A lateralization index (LI) was calculated to assess hemispheric dominance under each condition:

$$\begin{array}{l} \text{LI}=\left({\text{ERD}}_{\text{L}}-{\text{ERD}}_{\text{R}}\right)/{\text{ERD}}_{\text{L}}+{\text{ERD}}_{\text{R},} \end{array}$$

where ERD_R_ and ERD_L_ represent the overall ERD areas (%) (cerebral activation) of the CH (C4) and IH (C3) brain areas, respectively. An LI of 1 indicated total dominance of the non-active (mirrored) cortex and an LI of−1 indicated total dominance of the active cortex. One-way repeated-measure ANOVAs were used to assess the effect of time-phase across the three test conditions in the both groups. Significant models were assessed by Bonferroni *post-hoc* tests to compare differences between the time-phases.

Mauchly's test was used to test for violations of the assumption of sphericity in all ANOVA models. When the assumption of sphericity was rejected, adjustments were made using the Greenhouse-Geisser procedure.

## Results

### Mu Band Findings

The results of the four-way repeat-measures ANOVA showed that the group, the test condition, and the time phase were significant factors on the ERD areas (%) (*p* < 0.001). No significant four-factor and three-factor interactions were found. Interactions between two factors were only found for phase^*^group (*p* < 0.001) and phase^*^condition (*p* < 0.001). Since the brain area was not a significant factor and had not interactions with the other factors, we conducted three-way repeated-measures ANOVA of the other variables for each brain areas.

**Figure 3:**
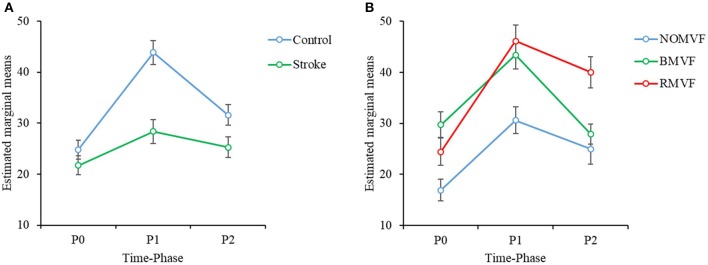
Three-way repeated-measures analysis of variance was used to examine the effects of group, test condition, and time-phase on the event-related desynchronization (ERD) areas (%) in the mu band. **(A)** There is a statistically significant interaction effect between group and time-phase in the IH brain area (C3). **(B)** There is a significant interaction effect between the test condition and time-phase in the CH brain area (C4).

The results of the three-way repeated-measures ANOVA showed that for each brain area, the effects of group, test-condition, and time-phase were significant (*p* < 0.005). No significant interactions were found for the SMA brain area (Cz), while significant interactions were found between time-phase and group for the IH brain area (C3) and between time-phase and test-condition for the CH brain area (C4). For the SMA brain area (Cz), *post-hoc* analyses revealed that the ERD area (%) was smaller in the stroke group than in the control group. The ERD areas (%) were largest at P1, second at P2, and the smallest at P0. The ERD areas (%) under the BMVF and RMVF conditions were significantly larger than that under the NoMVF condition (*p* < 0.001). For the IH brain area (C3), the ERD areas (%) of the stroke group were smaller than the control group during the three-time phases (Figure 3A). For the CH brain area (C4), the ERD area (%) under the RMVF condition was smaller than that under the BMVF condition at P0, but then were larger than those under the BMVF and NoMVF conditions at P1 and P2 (Figure 3B).

**Figure 4:**
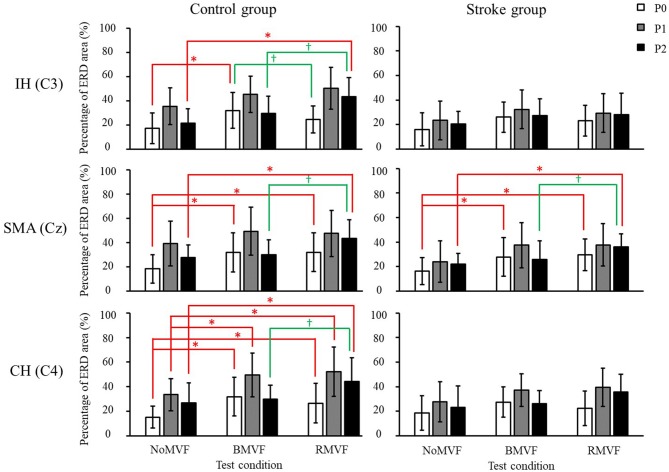
Mu band event-related desynchronization (ERD) areas (%) of the control and stroke group (*n* = 16 each) with error bars (standard deviations) for the three test conditions (NoMVF, BMVF, and delayed RMVF) for the IH (C3), SMA (Cz), and CH (C4) brain areas, respectively. The ERD areas (%) are shown as P0: white, P1: gray, and P2: black; a time-course change in cortical activation can be observed. ^*,^^†^ERD areas (%) of this test condition are significantly higher than the areas of the previous test condition for the same brain area.

Compare our results with the previous study, the results showed similar trends. The test-condition and time-phase were significant factors of the ERD area (%) for the two groups (*p* < 0.050) for the three brain areas, except the time phase for the IH brain area (C3). As Figure 4 shows, for the control group and for the three brain areas, the ERD areas (%) under the BMVF and RMVF conditions at P0 were mostly significantly larger than that under the NoMVF condition, and the ERD area (%) under the RMVF condition was significantly larger than those under the NoMVF and BMVF conditions at P2. For the stroke group, significant differences of the ERD areas (%) between three time-phase were only found in the SMA brain area (Cz). The same with the control group, the ERD area (%) under the RMVF condition was significantly larger than those under the NoMVF and BMVF conditions at P2.

**Figure 5:**
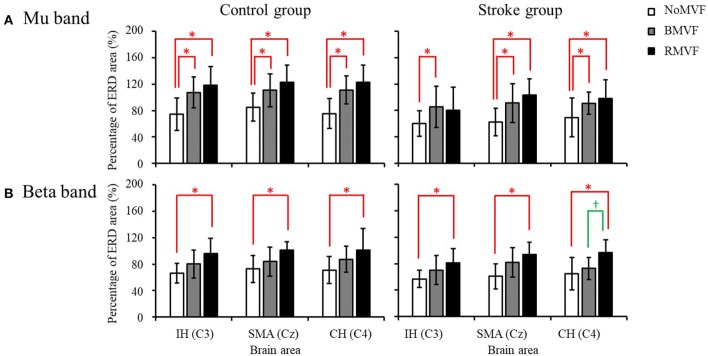
Overall event-related desynchronization (ERD) areas of the control and stroke group (*n* = 16 each) with error bars (standard deviations) for the three test conditions (NoMVF: white; BMVF: gray, and RMVF: black) for the IH (C3), SMA (Cz), and CH (C4) brain areas, respectively. **(A)** Overall ERD areas (%) of the mu band **(B)** Overall ERD areas (%) of the beta band. ^*,^^†^Overall ERD areas (%) of this test condition are significantly higher than the previous one for the same brain area.

The results of the overall ERD areas (%) found that for both groups, the ERD areas (%) under the RMVF and BMVF conditions were significantly larger than that in the NoMVF condition, except for the ERD area (%) under the RMVF condition in the IH brain area (C3). No significant differences were found between the ERD areas (%) under the BMVF and RMVF conditions (Figure 5).

### Beta Band Findings

**Figure 6:**
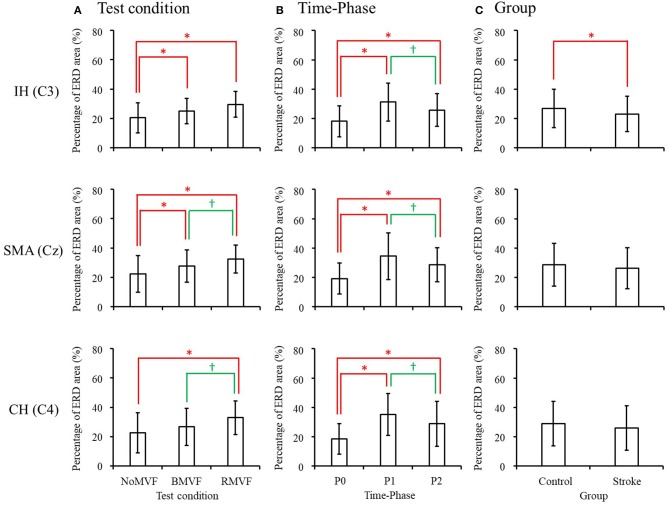
Three-way repeated-measures analysis of variance in the beta band revealed that both test condition and time-phase have main effects in all three brain areas, while group also had a main effect in the IH brain area (C3). Comparison of the mean value of event-related desynchronization (ERD) areas for each main effect are shown for **(A)** test condition, **(B)** time-phase, and **(C)** group. ^*,^^†^ERD areas (%) of this test condition, time-phase, or group are significantly higher than the previous one in each comparison.

The results in the beta band were similar to those in the mu band. The brain area had no interactions with the other variables, so we entered the other three variables (group, time-phase, and test-condition) into the three-way repeated measures ANOVAs for each brain area. The results revealed that the variables of group, test-condition, and the time phase were significant factors of the ERD areas (%) for the IH brain (C3). No interactions were found among the three variables. As Figure 6 shows, the ERD area (%) was smaller in the stroke group than that in the control group (*p* = 0.004), the ERD area (%) were largest at P1, P2 second, and P0 the smallest. The ERD areas (%) had no significance under the BMVF and RMVF conditions (*p* = 0.095), but were significantly larger than that under the NoMVF condition (*p* < 0.020). For the SMA (Cz) and CH (C4) brain areas, test-condition and time-phase but not group were significant factors (*p* < 0.001). No interactions were found among the three variables. For both brain areas, the ERD areas (%) were largest at P1, and the at P2, and smallest at P0. The ERD area (%) under the RMVF condition was significantly larger than that under the BMVF condition and NoMVF condition (*p* < 0.010).

The results of the overall ERD areas (%) analyses found that for both groups, the ERD area (%) under the RMVF conditions were significantly larger than that under the NoMVF condition for all brain areas. Moreover, the ERD area (%) under the RMVF condition was significantly larger than that under the BMVF condition (C4: 80.356 ± 19.733) in the stroke group for the CH brain area (C4) (Figure 5).

### Lateralization

Table 2 lists the LI values of the mu band and the beta band, and the significant differences among the three time-phases under each condition. No significant main effect of time-phase was shown in the mu band in the control group. On the other hand, in the stroke group, there was only a significant effect of time-phase under the RMVF condition (*p* = 0.044), indicating that the brain activity during P1 and P2 was significantly lateralized to the left hemisphere, as compared to P0 (*p* < 0.050) (Table 2). In the beta band, no significant differences in the ERD area (%) were found among the time-phases under the three conditions in both groups (*p* > 0.050).

**Table 2:** Lateralization analyses among the three conditions in the mu band and the beta band.

**Test condition**	**NoMVF**			**BMVF**			**RMVF**		
**Time-phase**	**P0**	**P1**	**P2**	**P0**	**P1**	**P2**	**P0**	**P1**	**P2**
**(A) MU BAND**									
Control group (*n* = 16)	−0.01	−0.02	0.07	−0.02	0.03	0.03	0.01	0.01	−0.01
Lateralization index	(0.46)	(0.27)	(0.47)	(0.30)	(0.24)	(0.33)	(0.33)	(0.23)	(0.32)
Stroke group (*n* = 16)	0.13	0.09	0.00	0.05	0.09	0.01	−0.04	0.19^*^	0.20^*^
Lateralization index	(0.46)	(0.39)	(0.38)	(0.32)	(0.29)	(0.37)	(0.42)	(0.31)	(0.38)
**(B) BETA BAND**									
Control group (*n* = 16)	0.00	0.02	0.05	0.09	0.02	0.03	−0.08	0.01	0.07
Lateralization index	(0.37)	(0.21)	(0.38)	(0.47)	(0.22)	(0.27)	(0.40)	(0.24)	(0.20)
Stroke group (*n* = 16)	0.08	0.09	−0.04	0.00	0.08	0.00	0.00	0.12	0.08
Lateralization index	(0.47)	(0.38)	(0.33)	(0.54)	(0.27)	(0.35)	(0.37)	(0.27)	(0.30)

*Data are shown as mean with standard deviations*.

*One-way repeated measures ANOVAs were employed to calculate statistical significance of time-phase under three conditions in each group*.

* *The percentage of ERD areas had statistically difference compared to the same phase of P0 in each condition (p < 0.05). No statistically significant difference was found in beta band*.

## Discussion

The present study was an extension of Lee et al.'s study (17). We used the same DMT system as in that study to manipulate MVF, but modified the tasks from pressing a button to grasping a paper cup. We then investigated the effects of the DMT system in both healthy subjects and stroke patients. The present study had three main findings. First, less cortical activation was found in stroke patients than in healthy subjects while executing the tasks. Second, in general, cortical activations were larger under both BMVF and RMVF conditions than under the NoMVF condition. Third, in general, the cortical activation tended to be larger under the RMVF condition than under the BMVF condition, especially at P2, although most of these differences did not reach statistical significance. The results of our study supported the notion that the new DMT system used in our study could be used in stroke patients and that RMVF training may benefit restoration of hand function in stroke patients.

Our study investigated the effect of mirrored and reciprocal movements on the mu and beta bands. According to Perry and Bentin's study, mu band suppression was higher in the active cup grasping condition than in the passive viewing condition (24). Our results shared the same effect of an active movements on the brain wave. As expected, the ERD areas (%) in the mu and beta bands in healthy subjects were generally significantly larger than those in stroke patients, which was in line with decreased cortical activation in stroke patients with respect to healthy subjects. A study by Stepien et al. (18) also investigated ERD in sensorimotor EEG rhythms in hemiparetic patients after an acute stroke and found decreased cortical neuronal network activity in both lesioned and intact hemispheres in stroke patients as compared to controls. Several magnetoencephalography studies have also reported that movement-related beta desynchronization in stroke patients is generally smaller than that in healthy subjects (19, 25, 26).

It was plausible that cortical activations were larger under the BMVF and RMVF conditions than under the NoMVF condition, since numerous studies have supported the efficacy of MT. Moreover, we found that the ERD area (%) in the mu band, in general, was not significantly different under the RMVF and BMVF conditions, contrary to our expectation. However, when we specifically inspected the effects of the test condition and time-phase on the ERD areas (%) in the mu band, in each group, as was done in Lee et al.'s study (17), we found that, for the healthy subjects, the ERD areas (%) under the RMVF condition was significantly larger than those under the NoMVF and BMVF conditions for all three brain areas at P2. The larger ERD area (%) found under the RMVF condition at P2 indicated that RMVF may reinitiate and prolong cortical activation in participants.

Similar trends for the ERD areas (%) in the mu band were found in stroke patients, but only in the SMA brain area (Cz). That is, the ERD areas (%) under the BMVF and RMVF conditions were significantly larger than that under the NoMVF condition at P0, and the ERD area (%) under the RMVF condition was significantly larger than those under the BMVF and NoMVF conditions at P_2_. However, we found that the ERD areas (%) across the three time-phases under the BMVF and RMVF conditions were also larger than those under the NoMVF condition at CH brain area (C4). This indicated a tendency for larger cortical activation in the contralateral primary sensorimotor hand areas with MVF, although the differences were not statistically significant. Taken together, brain activation patterns in stroke patients in the present study were similar to that in our healthy subjects, and those reported in healthy subjects by Lee et al. (17). Moreover, the results of the analysis of the overall ERD areas (%) in the beta band showed that the overall ERD area (%) under the RMVF condition was significantly larger than that under the BMVF condition in the stroke group in the CH brain area (C4). These results indicated that stroke patients may also benefit from RMVF training, because this training increased activation in the supplementary motor area of the brain. Therefore, this MT system may be useful in clinics for stroke patients undergoing MT. Furthermore, the results of our study indicated that the use of RMVF could facilitate cortical activation in both stroke patients and healthy subjects.

There are two factors that warrant further discussion and that might explain the non-significant differences in the ERD area (%) in the mu band in the IH (C3) and CH (C4) brain areas between stroke patients and healthy subjects. First, as mentioned above, brain activation was decreased in stroke patients because of their brain injury. Small magnitude changes in brain activation at each time-phase, particularly in the brain injury region, may have resulted in the non-significant differences in brain activation. Second, given that these participants had left hemisphere strokes, the central brain regions, such as the corpus callosum, may have functionally compensated for the damaged left hemisphere, resulting in greater activations at the SMA brain area (Cz), rather than at the IH brain area (C3) (27).

Consistent with Lee et al.'s study (17), our lateralization analyses revealed that, for the analyses in the mu band, cortical responses to MVF were more right-lateralized (the mirror side) in stroke patients (there was no significant lateralization in controls), and there were no significant differences in the analyses of the beta band in the stroke patients. Previous studies using the lateralized readiness-potential have shown lateralized activation in the sensorimotor area of the contralateral hemisphere in response to the MVF in MT in healthy controls (28). Bai et al. found that for right-handed healthy subjects, left brain activation while moving left fingers was greater than right activation while moving right fingers, especially in the P0 time phase (29). It means for a right-handed subject, the non-dominated hand movements require more bilateral activation than the dominated hand movements. This indicated that unfamiliar hand movement requires bilateral brain activation, where as familiar or well-practiced hand movements prone to using only one side of the brain. In our study, all subjects had right hemiparesis, and the movements of the affected hand could be viewed as unfamiliar movements, therefore requiring more lateralization process of the brain. However, our study found that this kind of activation was more prominent in the P1 and P2 stages rather than in the P0 stage as Bai and his colleague described. The possible explanation to this phenomenon was that the brain activation was slower and required more time for the stroke subjects than for the healthy subjects. Our study further found that lateralization was more salient with RMVF, suggesting that RMVF may activate the impaired side of the brain better than do the other MT conditions. Therefore, among the three visual feedback conditions tested, RMVF could be a better method for increasing stoke patients' brain activity.

The main difference between our study and Lee et al.'s study (17) was the design of the task, (i.e., grasping a paper cup versus pressing a button). As compared to pressing a button, grasping a cup is a more common daily activity and is thus more meaningful to many participants. Such a goal-oriented task might increase cortical activity and thus induce greater motor recovery. It has been reported that patients' upper extremity functioning may be improved and maintained by performing functional movements associated with tasks of daily living (30, 31). However, we did not compare differences in brain activity between these two tasks in this study, as this was not directly relevant to our study aims. Future studies may seek to identify brain activation differences across different tasks, which may serve as a reference for clinicians when implementing RMVF.

Although our study provided evidence for RMVF-mediated increases in brain activity in both healthy subjects and in stroke patients, several methodological issues should be addressed. First, as in Lee et al.'s study, the study environment and participants' seating position differed from those used previously. This was due to practical, equipment-related concerns. These differences may have caused low mu rhythm rates in our subject population (17). However, we also found that the ERD area (%) was sufficiently sensitive to detect the effects of a grasping movement and MVF in cortical areas. Second, large variations in the ERD areas (%) were revealed in the stroke patients assessed here. These may have been due to differing case severities and injury locations. However, the present study did not assess detailed brain injury information in patients. Therefore, we were unable to analyze brain activation signal changes further based on stroke severity. Given that the present study demonstrated that stroke patients might benefit from the RMVF condition, future studies should use RMVF across different types of stroke cases, with varying severity, to determine the interaction between stroke type/severity and RMVF-linked changes. This may further help clinicians specify the patient characteristics most indicative of RMVF-based intervention success.

In summary, our study used the novel DMT system to manipulate MVF for MT and investigated its effects in both healthy subjects and stroke patients. Our study findings indicated that, generally, RMVF resulted in larger brain activations during MT in both healthy subjects and stroke patients. The lateralization index shows significant difference during the RMVF condition, where the data of P1 and P2 in the stroke group were both greater than P0. Therefore, RMVF training using DMT system compared to conventional MT enhances cortical activation particularly in the SMA (Cz), and can be applied for stroke patient therapy.

## Data Availability Statement

The raw data supporting the conclusions of this manuscript will be made available by the authors, without undue reservation, to any qualified researcher.

## Ethics Statement

The protocol was carried out in accordance with the recommendation of Kaohsiung Cheng Kung Memorial Hospital IRB committee.

## Author Contributions

H-ML conceived of the study, developed the digital mirror system. P-CL participated in the analysis and interpretation of the results, and wrote the manuscript. C-SC participated in the development of the digital mirror system, and helped revised the manuscript. Y-YL helped recruit the patients for testing, and aided in the data collection and in the interpretation of findings. C-LC and W-CC helped to recruit the subjects for testing and performed the statistical analyses. All authors read and approved the final manuscript.

### Conflict of Interest

The authors declare that the research was conducted in the absence of any commercial or financial relationships that could be construed as a potential conflict of interest.
